# Co-Amorphization of Kanamycin with Amino Acids Improves Aerosolization

**DOI:** 10.3390/pharmaceutics12080715

**Published:** 2020-07-30

**Authors:** Bishal Raj Adhikari, Kārlis Bērziņš, Sara J. Fraser-Miller, Keith C. Gordon, Shyamal C. Das

**Affiliations:** 1 School of Pharmacy, University of Otago, Dunedin 9054, New Zealand; adhbi024@student.otago.ac.nz; 2 The Dodd-Walls Centre for Photonic and Quantum Technologies, Department of Chemistry, University of Otago, Dunedin 9016, New Zealand; berka456@student.otago.ac.nz (K.B.); sara.miller@otago.ac.nz (S.J.F.-M.); keith.gordon@otago.ac.nz (K.C.G.)

**Keywords:** dry powder, co-amorphous, stability, aerosolization, kanamycin, amino acids, low-frequency Raman spectroscopy, principal component analysis

## Abstract

Different formulation techniques have been investigated to prepare highly aerosolizable dry powders to deliver a high dose of antibiotics to the lung for treating local infections. In this study, we investigated the influence of the co-amorphization of a model drug, kanamycin, with selected amino acids (valine, methionine, phenylalanine, and tryptophan) by co-spray drying on its aerosolization. The co-amorphicity was confirmed by thermal technique. The physical stability was monitored using low-frequency Raman spectroscopy coupled with principal component analysis. Except for the kanamycin-valine formulation, all the formulations offered improved fine particle fraction (FPF) with the highest FPF of 84% achieved for the kanamycin-methionine formulation. All the co-amorphous formulations were physically stable for 28 days at low relative humidity (25 °C/<15% RH) and exhibited stable aerosolization. At higher RH (53%), even though methionine transformed into its crystalline counterpart, the kanamycin-methionine formulation offered the best aerosolization stability without any decrease in FPF. While further studies are warranted to reveal the underlying mechanism, this study reports that the co-amorphization of kanamycin with amino acids, especially with methionine, has the potential to be developed as a high dose kanamycin dry powder formulation.

## 1. Introduction

Pulmonary drug delivery to treat lung infections is an important area of research [1,2]. Achieving a high drug concentration at lower doses and reducing associated side effects are the advantages of the local delivery of drugs to the lungs [3,4]. Nebulizers, metered-dose inhalers, and dry powder inhalers are the different types of devices used for pulmonary drug delivery [5]. In order to deliver the dose of antibiotics that is required for treating lung infections, dry powder inhalers are the choice of devices [6]. Moreover, dry powder inhalers offer advantages in terms of product stability and ease of administration [2,7]. Dry powders can be prepared using various methods, such as solvent-mediated processes, milling or spray drying [8,9]. Regardless of the involved preparation method, an attribute of high particle fraction with size less than 5 microns is desired in the dry powder formulations to achieve deep lung delivery [10].

Spray drying has emerged as a promising technique for the preparation of dry powder due to its scalability, reproducibility as well as efficient particle engineering capability [11]. However, in many cases, spray-dried powders show poor aerosolization, and a plethora of work has been reported on the use of different molecules (excipients/drugs) to improve aerosol performance. Examples of excipients that have been used to enhance aerosol performance include amino acids [12], sodium stearate [13], and phospholipids [14]. Among these, amino acids have been extensively utilized with the assumption that, being endogenous molecules, they would have the least toxicity concern for pulmonary drug delivery compared to other excipients [15]. However, caution should be maintained in a population with amino acid metabolic disorder. For example, the inhalation of dry powder with phenylalanine in patients with phenylketonuria could be a safety concern [16]. Leucine is one of the most commonly used amino acids. It is a hydrophobic amino acid that has been claimed to reduce moisture sorption and particle adhesion by surface enrichment of composite particles produced by spray drying [17]. Our research group has previously reported on the use of leucine to improve fine particle fraction (FPF) of kanamycin [18].

However, the use of alternative amino acids to achieve improved aerosolization is limited. The other amino acids that have been trialed as an aerosolization enhancer include methionine, phenylalanine, asparagine, tryptophan, arginine, histidine, threonine, glycine, and lysine [19,20,21,22,23,24]. Amino acid-based co-amorphous systems have recently been introduced as a potential strategy to improve the stability of amorphous material [25,26]. This technique is also being explored to improve aerosol performance of inhalable spray-dried particles. For example, the aerosolization of budesonide-arginine co-amorphous spray-dried powder was higher than budesonide alone [22]. However, these particles were not prepared using the same experimental conditions (solvent system and spray dryer operating parameters). Spray-dried particles prepared from different solvent systems or feed concentrations are known to show varying aerodynamic behavior, hence, it would only be speculative to assume that the observed aerosolization improvement in these co-amorphous spray-dried particles is due to co-amorphization with amino acid [27,28,29]. Similarly, inhalable simvastatin-lysine and simvastatin-tryptophan co-amorphous spray-dried particles have also been reported; however, the aerosolization of formulation containing only simvastatin has not been discussed for comparison [21].

**Figure 1:**
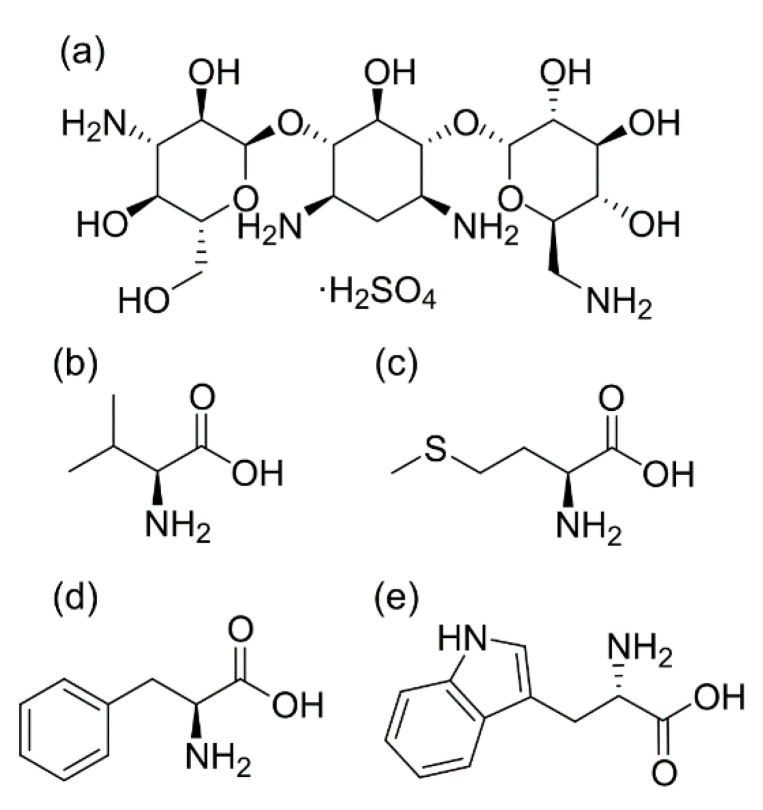
Chemical structures of (**a**) kanamycin sulfate, (**b**) L-valine, (**c**) L-methionine, (**d**) L-phenylalanine, and (**e**) L-tryptophan.

The present study aimed at elucidating the influence of co-amorphization of a drug with different amino acids on aerosolization. Kanamycin (as sulfate) (Figure 1), an anti-tubercular drug, was used as a model drug [18]. Four different amino acids (valine, methionine, phenylalanine, and tryptophan) were chosen for the preparation of potential inhalable co-amorphous particles using spray drying (Figure 1). The amino acids were selected based on their van der Waals volume, hydrophobicity, and their ability to form co-amorphous systems [30,31,32]. Solvent system, the concentration of kanamycin in feed solutions, and spray drying conditions were kept constant during the preparation of all the formulations to reduce variables affecting aerosolization. Furthermore, the stability of the co-amorphous spray-dried particles under different stressed conditions of temperature/humidity and its implications for aerosolization were also scrutinized. In particular, Raman spectroscopy was applied to investigate the transformation behavior of the particles in conjunction with principal component analysis.

## 2. Materials and Methods

### 2.1. Materials

Kanamycin sulfate (molar mass- 582.6 g/mol, ≥99% purity) was purchased from Hangzhou Danyanchem Limited, Hangzhouzhejiang, China. All amino acids (L-valine, L-methionine, L-phenylalanine, and L-tryptophan), phenyl isocyanate (PIC), silicon oil, trimethylamine (TEA) used were of reagent grade and were purchased from Sigma-Aldrich, MI, USA. Size 3 hydroxypropyl methylcellulose (HPMC) capsules were generously provided by Capsugel Japan Inc., Kanagawa, Japan. Acetonitrile (high-performance liquid chromatography grade) was purchased from Merck, Darmstadt, Germany. Purified water was obtained using Millipore continental water systems (Millipore Corporation, Burlington, MA, USA).

### 2.2. Preparation of Spray-Dried Formulations

Kanamycin sulfate was spray dried with different amino acids (valine, methionine, phenylalanine, and tryptophan) at a 1:1 molar ratio. Kanamycin sulfate (1500 mg) and an equivalent amount of amino acid (amount to create 1:1 molar ratio with kanamycin) were dissolved in 750 mL of purified water (Table 1). All feed solutions were further filtered through a 0.45 µm nylon filter before spray drying using a Buchi B-290 Mini Spray Dryer (Buchi Labortechnik AG, Flawil, Switzerland). The spray drying conditions were established based on a previous work on kanamycin from our research group [18]. The aspiration rate and feed rate were fixed at 100% and 2 mL/min, respectively, while the inlet temperature was set at 170 °C. The solutions were spray dried using a nozzle with 0.7 mm diameter in drying gas (air) with a flow rate of 670 L/h. An outlet temperature of 100 ± 2 °C was recorded. All the obtained samples were transferred to screw-capped glass vials, sealed with paraffin film, and stored in a desiccator (with silica gel) at room temperature. The yield of the formulations (%) was calculated by simply dividing the amount of powder that could be transferred to a glass vial by the amount of solid (drug and amino acid) initially taken for preparation of feed solution.

### 2.3. Powder X-ray Diffraction (PXRD)

The powder X-ray diffraction (PXRD) patterns were recorded using an X’Pert PRO MPD PW3040/60 X-ray diffractometer (Malvern Panalytical, Malvern, UK) using Cu K*α* radiation and equipped with a rapid real-time multi-strip (RTMS) X’Celerator detector. Samples were analyzed over a 2θ range of 5–35° at a rate of 6°/min. The data were collected using an X’Pert Data Collector (Malvern Panalytical) and analyzed using the HighScore suite (Malvern Panalytical).

### 2.4. Thermogravimetric Analysis (TGA)

The water content in each spray-dried formulation was determined using a Q550 TGA (TA Instruments, New Castle, DE, USA). Approximately 5 mg of sample was heated, in a platinum pan, at a rate of 10 °C/min from room temperature (~25 °C) to 200–300 °C under a nitrogen purge of 40 mL/min. The data were acquired and analyzed using TRIOS software (TA Instruments, New Castle, DE, USA).

### 2.5. Modulated Differential Scanning Calorimetry (MDSC)

A measured quantity of 2 ± 0.1 mg of spray-dried powder was crimped in a non-hermetic aluminum pan. The powder was then heated at a rate of 5 °C/min from −20 to 150 °C with a modulation period of 40 s and a modulation amplitude of ±0.53 °C under nitrogen flow (50 mL/min) using a Q500 DSC (TA Instruments, New Castle, DE, USA). The temperature scale and heat flow of the instrument were calibrated using indium as standard. The data were analyzed using TRIOS software (TA Instruments, New Castle, DE, USA). The glass transition temperature (*T**_g_***) was taken as the midpoint of the step change in reversing heat flow of the sample.

Theoretical *T**_g daa_*** of the kanamycin-tryptophan co-amorphous system was calculated using the Gordon-Taylor (GT) Equation [33,34]:

*T**_g daa_*** = (W_d._*T_gd_* + K.W_aa._T_gaa_)/(W_d_ + K.W_aa_) (1)

where:

W_d_ = weight fraction of the drug;

W_aa_ = weight fraction of the amino acid;

*T_gd_* = *T**_g_*** of the drug (°C);

T_gaa_ = *T**_g_*** of the amino acid (°C);

K = constant;

K = (*T_gd_* D_d_)/(T_gaa_ D_aa_).

where:

D_d_ = density of the drug (g/cm^3^); 

D_aa_ = density of the amino acid (g/cm^3^).

The density of kanamycin was taken as 0.67 g/cm^3^, while the density of tryptophan was taken as 1.30 g/cm^3^ [18,35].

### 2.6. Attenuated Total Reflectance-Fourier Transform Infrared (ATR-FTIR) Spectroscopy

Fourier Transform Infrared (FTIR) spectra were recorded by placing approximately 2 mg of the sample on the GladiATR accessory (Pike Technologies, Madison, USA) equipped in a Varian 3100 FTIR spectrometer (Varian Inc., California, CA, USA). The spectra were collected at a 4 cm^−1^ resolution with 64 scans over a range of 400–4000 cm^−1^ using Resolution Pro software (Agilent Technologies Inc., California, CA, USA). Peak positions were also identified using the same software.

### 2.7. Low-Frequency Raman (LFR) Spectroscopy

The Low-Frequency Raman (LFR) measurements were carried out using a custom-built system [36] with an excitation source from a 785 nm laser module (Ondax Inc., Monrovia, CA, USA) that was filtered by BragGrate band pass filters (OptiGrate Corp., Oviedo, FL, USA) to remove amplified spontaneous emission before irradiating the sample. Backscattered light from the sample was collected and filtered through a set of volume Bragg gratings (Ondax Inc., Monrovia, CA, USA) and focused into a LS 785 spectrograph (Princeton Instruments, Trenton, NJ, USA) via a fiber-optic cable. The light was dispersed onto a CCD detector (PIXIS 100 BR CCD, Princeton Instruments, Trenton, NJ, USA) and the data were calibrated using a sulfur, 1,4 bis (2-methylstyryl) benzene (BMB), and a toluene and acetonitrile solvent (1:1) standards. Spectra were collected over the spectral window −360 to 2030 cm^−1^ with a 5–7 cm^−1^ resolution. Each spectrum was averaged from 60 scans with an integration time of 1 s, and the sample spot size was approximately 500 μm. Specific spectral range (900–1000 cm^−1^) was used for the band analysis, and fitted peak positions were determined using the peak find function (default settings) in the SpectraGryph 1.2.14. software [37].

### 2.8. Computational Details

Theoretical solid-state density functional theory (DFT) calculations were performed using the fully periodic CRYSTAL14 software package [38] to primarily model the full Raman spectrum (including the low-energy phonon modes) of kanamycin sulfate monohydrate. For this purpose, B3LYP hybrid functional [39] with van der Waals interactions treated according to the D2 method of Grimme was employed [40]. C, N, O, and S atoms were described by the m-6-311G(d) split valance, triple-ζ basis set [41], whereas H atoms were represented using the 7-311G [42] basis set. For the input files, all the basis sets were directly adapted from the CRYSTAL library. The crystal structure of kanamycin sulfate monohydrate (CCDC reference: 610593) [43] was optimized with implied restrictions on lattice parameters as initially performed full relaxation caused ambiguities for the vibrational analysis. The dielectric tensor and Raman intensities were calculated analytically using the coupled-perturbed Hartree-Fock/Kohn-Sham (CPHF/CPKS) approach [44]. Energy convergence criteria was set to ΔE ≤ 10^−8^ and 10^−10^ hartree for the geometry optimization and vibrational calculations, respectively. The calculated vibrational modes were visualized using the MOLDRAW 2.0 (version H1) software.

### 2.9. Scanning Electron Microscopy (SEM)

The morphology of the spray-dried particles was examined using Zeiss Sigma scanning electron microscopy (SEM) (Carl Zeiss Inc., Oberkochen, Germany). Using a simple dusting method, the powder was spread on adhesive carbon tape. The particles were then coated with a very fine layer of gold/palladium alloy (80:20 ^w^/_w_) using a Quorum Q150TE turbo-pumped carbon coater (Quorum Technologies Ltd., Sussex, UK). Images were captured at an accelerating voltage of 5 kV. An image processing software, ImageJ (National Institutes of Health, Bethesda, MD, USA), was used to determine the average geometric diameter of the particles. Diameters of more than 300 particles were measured for each sample.

### 2.10. In-Vitro Aerosolization Study

The in-vitro aerosolization behavior of spray-dried particles containing kanamycin only and co-amorphous spray-dried particles were assessed by a Next-Generation Impactor (NGI) (Copley Scientific Limited, Nottingham, UK). Airflow for dispersion was created using a Copley HCP5 vacuum pump while the flow was adjusted to 100 L/min using a Copley TPL 2000 critical flow controller and an electronic flow meter supplied by the manufacturer. A thin layer of silicone oil with a viscosity of 10^−5^ m^2^/s (at 25 °C) was applied on all the stage plates to mimic thin fluid lining on the lungs to avoid undesired particle bounce. For each experiment, size 3 HPMC capsules were loaded with approximately 20 mg of the sample before activating them using a Foradil aerolizer (Novartis Pharmaceutical Limited, UK). The aerolizer with punctured/activated capsule was then carefully fitted to the mouthpiece (MP) on the induction port (IP), and the powder in the capsule was then dispersed under an inlet flow rate of 100 L/min for 2.4 s. Following actuation, the powder deposited at different stages/sections of the NGI was collected by rinsing them with purified water. These solutions were further analyzed using liquid chromatography to quantify dispersion in different stages/sections, which include aerolizer/capsule (AERO-CAP), MP, IP, stage 1 (S1), stage 2 (S2), stage 3 (S3), stage 4 (S4), stage 5 (S5), stage 6 (S6), stage 7 (S7) and micro-orifice collector (MOC). At an impactor inlet flow rate of 100 L/min, the cut size (D50) for S1–7 are 6.12, 3.42, 2.18, 1.31, 0.72, 0.40, and 0.24 µm, respectively [45]. Different in-vitro aerosolization parameters such as recovered dose (RD), percentage recovered dose (RD (%)), emitted dose (ED), percentage emitted dose (ED (%)), fine particle dose (FPD), and fine particle fraction (FPF), respirable fraction-3µm (RF_3µm_) were calculated. The RD referred to the total amount of drugs collected from aerolizer, MP, IP, and different stages of NGI. The RD (%) was calculated relative to the total amount of the drug that was initially loaded in capsules. The ED referred to the total amount of drug that left the aerolizer. The ED (%) was expressed relative to RD. The FPD was the amount of drug in the particles with aerodynamic diameter ≤5 µm (calculated by interpolation of the NGI data graph (cumulative mass vs. D50)) [46]. Similarly, RF_3µm_ was also calculated in a similar manner. Both the FPF and RF_3µm_ were expressed relative to ED.

### 2.11. High-Performance Liquid Chromatography (HPLC) Analysis

Kanamycin was quantified using a validated HPLC method [18,47]. Chromatography was performed using an LC-20AD HPLC (Shimadzu, Kyoto, Japan) equipped with an SPD-M20A photodiode array detector. A mixture of water (65%) and acetonitrile (35%) was used as mobile phase under an isocratic condition with a flow rate of 1.2 mL/min. The volume of injection was 20 µL for each sample, and a C18 Synergi Fusion Column (250 mm × 4.6 mm, 5 μm, 80 Å, Phenomenex, CA, CA, USA) fitted with a security guard column (4.0 mm × 3.0 mm, Phenomenex, California, USA) was used. Derivatization was conducted by adding 0.5 mL of kanamycin solution to a fresh 0.5 mL solution prepared by mixing 0.25 mL 0.5% trimethylamine solution (prepared in acetonitrile) and 0.25 mL 0.5% PIC (prepared in acetonitrile). This final solution was then heated in a water bath maintained at 70 °C for 10 min and cooled under running water before being analyzed using HPLC at 240 nm. The run time was 20 min and the retention time for the peak of interest due to the derivative was at ~10 min. The derivatization was specific for kanamycin and the derivative was stable for over 24 h [47]. Amino acids did not have any interference in the quantification of kanamycin. The calibration curve plotted using standard solutions of concentrations- 5, 12.5, 25, 50, and 100 µg/mL was linear (R^2^ ≥ 0.99) (Figure S1). The limit of detection (LOD) was 0.63 µg/mL and the limit of quantification (LOQ) was 1.93 µg/mL.

### 2.12. Stability Study

The stability of spray-dried particles was further assessed using three different conditions—25 °C/<15% relative humidity (RH), 25 °C/53% RH and 40 °C/75% RH for 28 days. Low-humidity (<15%) chambers were created using silica gel. An intermediate-humidity chamber (53% RH) was prepared using a saturated solution of magnesium nitrate, while a high-humidity chamber (75% RH) was prepared using a saturated solution of sodium chloride [48]. Approximately 300 mg of each formulation was spread in a petri dish and stored in the respective chambers. Initial characterization (day 0) of all the formulations was done using XRD, LFR, TGA, DSC, SEM, and NGI. Amorphicity (LFR), aerosolization (NGI), water content (TGA), and morphology (SEM) were again assessed at the end of 28 days. 

To gain insight into the stability of the formulations, Raman spectroscopy was explored with extensive sampling at 0, 3, 6, 9, 24, 33, 48, 57, 72 h, day 6, day 8, day 12, day 15, day 21, day 28, day 60, and day 90. In any condition, sampling was stopped if no spectral change (complete crystallization) was observed for consecutive time-points.

### 2.13. Statistical Analysis

Statistical analysis was performed using one-way analysis of variance (ANOVA) where applicable. The Newman-Keuls test was used as a post hoc test with a confidence level of 95% (*p* < 0.05) using DescTools package in R studio [49,50].

### 2.14. Principal Component Analysis (PCA)

PCA on the LFR data was done in Unscrambler V11.0 (Camo Analytics, Oslo, Norway). The spectra were preprocessed using a baseline offset followed by standard normal variate transformation of the spectral region 15 to 300 cm^−1^. PCA was calculated for each formulation separately. The NIPALS algorithm was applied with random cross validation where 2–3 spectra (representative of 2–3 samples) were removed with each PCA calculation for testing, which was repeated 20 times (20 segments of 2–3 samples). The first 2–3 PCs described >88% of the explained spectral variance and were used to examine the changes in the samples during storage.

## 3. Results and Discussion

### 3.1. Preparation of the Spray-Dried Formulations

Five different spray-dried formulations (kanamycin only (KO), kanamycin-valine (KV), kanamycin-methionine (KM), kanamycin-phenylalanine (KP), and kanamycin-tryptophan (KT)) were prepared using spray drying. The process yield was ~70% for all formulations (Table 1). In general, except valine, all the other amino acids improved the process yield.

### 3.2. Characterization of the Spray-Dried Formulations

The formulations were characterized using thermal analysis and spectroscopic methods.

#### 3.2.1. Amorphicity of the Formulations

**Figure 2:**
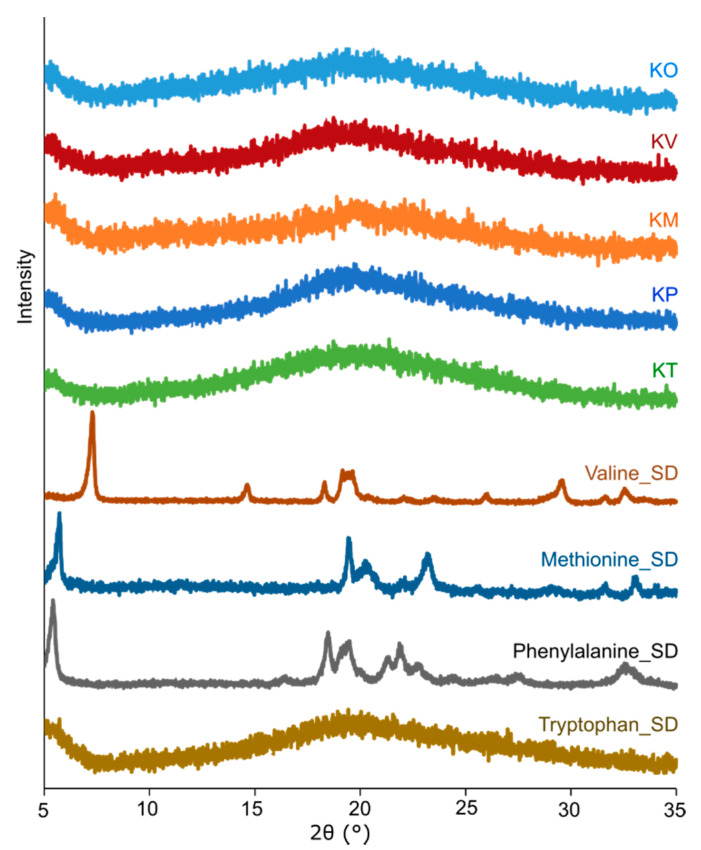
Representative X-ray diffractograms (XRD) of the spray-dried formulations (kanamycin only (KO), kanamycin-valine (KV), kanamycin-methionine (KM), kanamycin-phenylalanine (KP), and kanamycin-tryptophan (KT)) and spray-dried (SD) amino acids (methionine (Methionine_SD), valine (Valine_SD), phenylalanine (Phenylalanine_SD), and tryptophan (Tryptophan_SD)).

Four different amorphous kanamycin-amino acid systems (kanamycin-valine (KV), kanamycin-methionine (KM), kanamycin-phenylalanine (KP), and kanamycin-tryptophan (KT)) were produced by co-spray drying, as evidenced by the PXRD data (Figure 2). Kanamycin itself produced amorphous particles (KO) when spray dried alone, which was consistent with our previous study [18]. When amino acids were spray dried alone, all but tryptophan precipitated in crystalline form suggesting the possibility of a homogeneous amorphous matrix. Amorphicity was also confirmed using FTIR and Raman spectroscopy (Section 3.2.4).

#### 3.2.2. Water Content

All the spray-dried particles possessed 5 to 9% water (w/w; Table 2). The TGA thermograms are shown in Figure S2. Upon heating at a constant heating rate of 10 °C/min, there was a gradual loss in weight up to around 100 °C, which corresponds to the presence of water. The water could be residual or from the atmosphere, as amorphous matrices are relatively hygroscopic and known to sorb water via non-covalent interactions [51,52]. The co-amorphous systems had lower water content compared to the pure drug. The observation aligned well with the amino acids used. Such amino acids are relatively hydrophobic molecules, and, thus, they can be expected to create a more hydrophobic matrix.

#### 3.2.3. Determination of the Glass Transition Temperature (T_g_)

Conventional DSC showed endotherms in which the *T*_g_ of the amorphous material and dehydration were convoluted (Figure S3A). Therefore, modulated DSC was used to disentangle these concurrent events (Figures S3B and S4) [53,54]. The *T*_g_ of the samples are shown in Table 2. The fact that the mixtures show single *T*_g_ is consistent with a co-amorphous form. The experimental *T*_g_ of the KT co-amorphous system deviated from the *T*_g_ (~100 °C) calculated using the GT equation. Other drug-amino acid co-amorphous systems have also been reported to have *T*_g_ similar to that of the drug itself. For example, co-amorphization with methionine did not dramatically change the *T*_g_ of carvediol (*T*_g_ of the pure drug −38.0°; *T*_g_ of the co-amorphous form −38.7 °C) and mebendazole (*T*_g_ of the pure drug −110.5 °C; *T*_g_ of the co-amorphous form −109.3 °C) [26]. The observed negative deviation of the KT co-amorphous system from the GT equation might be due to non-ideal volume mixing [33,55]. No exothermic event (recrystallization of amino acid or kanamycin) in any of the MDSC thermograms suggested that the physical stability of the co-amorphous systems is likely not subject to change in temperature.

#### 3.2.4. Spectroscopic Assessment of Co-Amorphous Formulations

The FTIR spectra of kanamycin showed characteristic peaks corresponding to different functional groups/moieties. Bands were observed at ~1600 cm^−1^ (asymmetric bending) and ~1500 cm^−1^ (symmetric bending) from primary amine [56,57]. Weak bands at ~1340 cm^−1^ likely correspond to C-H bend. A strong broad band from 1200 cm^−1^ to 900 cm^−1^ with a peak at ~1020 cm^−1^ possibly encompasses multiple vibrations, particularly, sulfate ion (major), C-N stretch, cyclic ether (C-O-C), cyclohexane ring vibration, and C-O stretch [58,59]. Besides, the band at 601 cm^−1^ likely corresponds to sulfate ion bending [59].

The LFR spectra of kanamycin exhibited a series of peaks, consistent with crystalline LFR phonon modes. Some of the major peaks were at around 40, 85, and 107 cm^−1^. Based on the theoretical calculations, all of the aforementioned modes were found to encompass complex torsion motions, which was consistent with the presence of the complex network of intermolecular interactions, including a number of different hydrogen-bonding patterns (Figure S5) [43].

**Figure 3:**
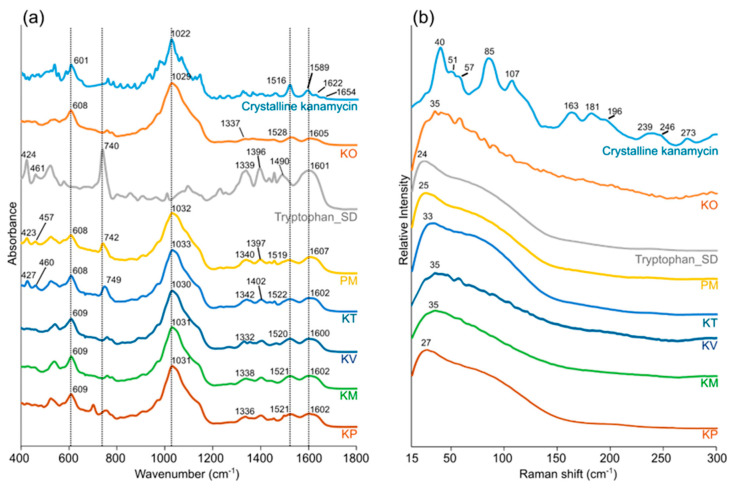
Representative (**a**) Fourier Transform Infrared (FTIR) and (**b**) Low-Frequency Raman (LFR) spectra of the different particles. From top to bottom: Crystalline kanamycin, kanamycin only formulation (KO), spray-dried tryptophan (Tryptophan_SD), a physical mixture (1:1) of amorphous kanamycin and amorphous tryptophan (PM), kanamycin-tryptophan formulation (KT), kanamycin-valine formulation (KV), kanamycin-methionine formulation (KM), and kanamycin-phenylalanine formulation (KP).

The spectra of a co-amorphous system and its respective physical mixture (amorphous kanamycin/amorphous amino acid) were compared to identify possible interactions. This assessment was focused around a kanamycin-tryptophan co-amorphous system, as tryptophan was the only amino acid successfully produced in the amorphous form. The FTIR spectra of the kanamycin-tryptophan co-amorphous system and physical mixture of amorphous kanamycin and amorphous tryptophan closely resembled each other. Subtle differences in band positions were observed (Figure 3a, Table S1), particularly at ~740 (Benzene/pyrrole ring deformation vibrations from tryptophan) [60], and ~1600 cm^−1^ (asymmetric bending of the amino group). The LFR spectra of these systems were also examined. In all instances, the spray-dried drug-amino acid formulations gave broad smooth low-frequency Raman spectra, consistent with amorphous systems [61,62]. Subtle differences were observed between the different formulations with small variations in the shape of the vibrational density of states (VDOS) and the position of the Boson peak. It is important to note that the KO, KV and KM formulations are not semi crystalline, the series of small peaks observed in Figure 3b are associated with the rotational side bands of air. These are detected when the analyte exhibits weak LFR signal.

#### 3.2.5. Particle Morphology and Size

**Figure 4:**
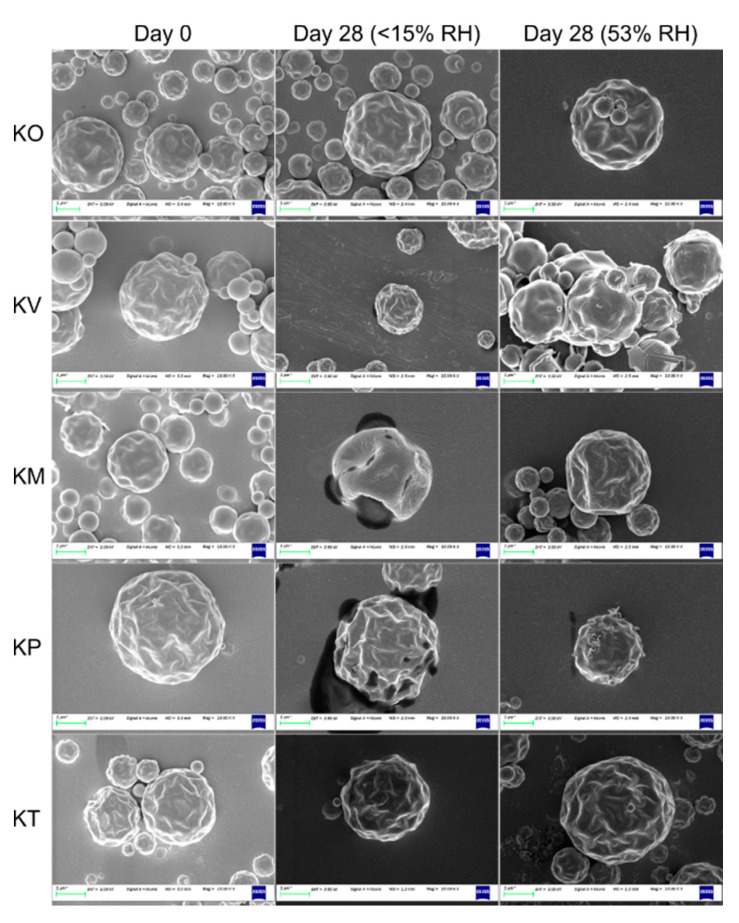
Representative SEM images of the formulations (kanamycin only (KO), kanamycin-valine (KV), kanamycin-methionine (KM), kanamycin-phenylalanine (KP), and kanamycin-tryptophan (KT)) on day 0 and day 28 when stored at 25 °C/<15% RH and 25 °C/53% RH.

Kanamycin formed spherical particles with pits and ridges on the surface upon spray drying (Figure 4). The presence of pits/ridges as well as collapsed structures suggested that the particles were hollow [29]. In general, all the kanamycin-amino acid co-amorphous particles were very similar to kanamycin spray-dried particles. However, the pits/ridges were slightly more prominent in the co-amorphous particles. Small agglomerates were observed in all formulations (Figure S6). The particles were ~1 µm in size for all the formulations (Table S2).

#### 3.2.6. In-Vitro Aerosolization

All the formulations (except KV) offered improved FPF (*p* < 0.05) compared to the FPF of KO formulation. However, RF_3µm_ was significantly improved by all the co-amorphous formulations and they had a lower particle deposition in MP/IP as well as stage 1 (Figure S7). Methionine was the most influential in improving the FPF and RF_3µm_. It increased the FPF and RF_3µm_ of kanamycin by 18 and 24%, respectively. A summary for these results has been presented in Table 3.

Particle deposition behavior is governed by a complex reciprocity, primarily among particle size [63], morphology [64], density [65], hygroscopicity [66], and surface energy [67,68]. Inclusion of amino acid to any formulation can modulate one or more of these factors and have a diverse effect on aerosolization. Surface enrichment induced surface asperities and decreased surface energy have been attributed as the major underlying mechanisms behind aerosolization enhancement by leucine [19,69]. The same was true for kanamycin-leucine formulation [18]. However, for other amino acids, it has not been well established. For example, methionine failed to improve the aerosolization of disodium chromoglycate [19]; however, it significantly improved the aerosolization of kanamycin in our study, suggesting that the ability of an amino acid to induce aerosolization enhancement to a drug/entity is not universal but idiosyncratic. Unlike with leucine, striking change in surface geometry was not observed with valine, methionine, phenylalanine, and tryptophan likely due to formation of co-amorphous matrix. Nevertheless, enhancement in aerosolization (FPF and/or RF_3µm_) was evident with the use of these amino acids. Here, as all the formulations had a similar particle size, the co-amorphous matrix or the change in surface asperities, even though tenuous, likely increased the dispersion of the particles into agglomerates (<5 microns) or primary particles by lowering cohesive forces and/or contact area between particles, as evidenced by the varying FPF and/or RF_3µm_ of the co-amorphous formulations. Further investigation is warranted to precisely understand the underlying mechanisms, as aerosolization behavior is influenced by multiple factors.

To the best of our knowledge, this is the first time successful aerosolization enhancement using methionine, valine (only RF_3µm_), and tryptophan has been reported. Moreover, we report the first case in which drug-amino acid co-amorphous spray-dried particles offered enhanced aerosol performance compared to spray-dried particles containing the drug alone when the particles were prepared using the same solvent system and spray drying conditions.

### 3.3. Stability Study

The aerosolization and physical stability of the formulations were evaluated using NGI and Raman spectroscopy, respectively. PCA was performed on the LFR spectra collected from the samples kept at different conditions over 3 months to visualize the subtle differences in the spectra over time. The criteria for choosing the relevant number of components were based on the explained variance and deviation between the calculation and cross validation, the magnitude of changes being described with each PC and how relevant is it for inclusion via examination of the loadings to check that they make spectroscopic sense. In all instances the first 2–3 PCs were selected.

**Figure 5:**
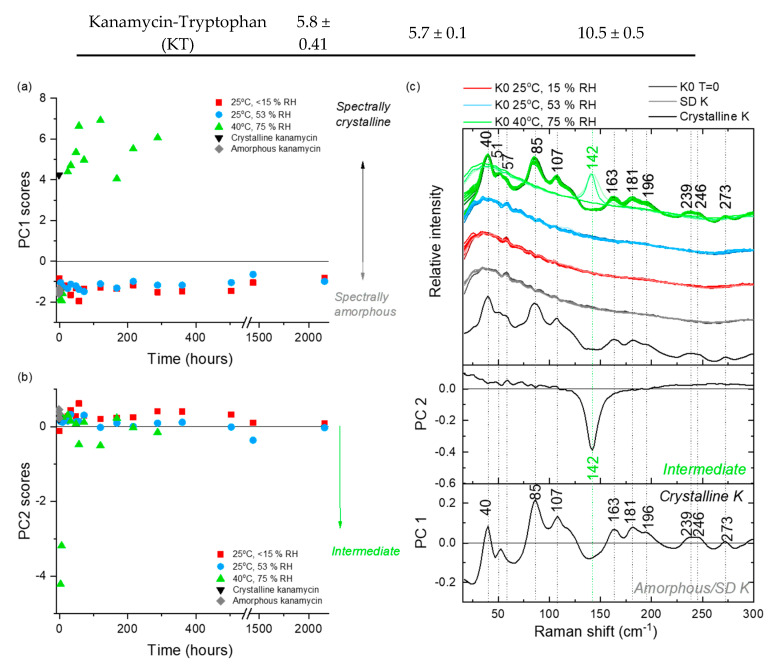
PCA analysis of the LFR spectra collected from KO samples stored under three different conditions (25 °C/<15% RH, 25 °C/53% RH and 40 °C/75% RH) over time. (**a**) PC 1 scores versus time, (**b**) PC 2 scores versus time and (**c**) loadings with comparative spectra. PC 1 accounts for 88% of the explained spectral variance and PC 2 accounts for a further 8% explained variance. Spectra from the different storage conditions in (**c**) have slight color graduations to highlight early (lighter coloring) versus latter (darker coloring) time points.

**Figure 6:**
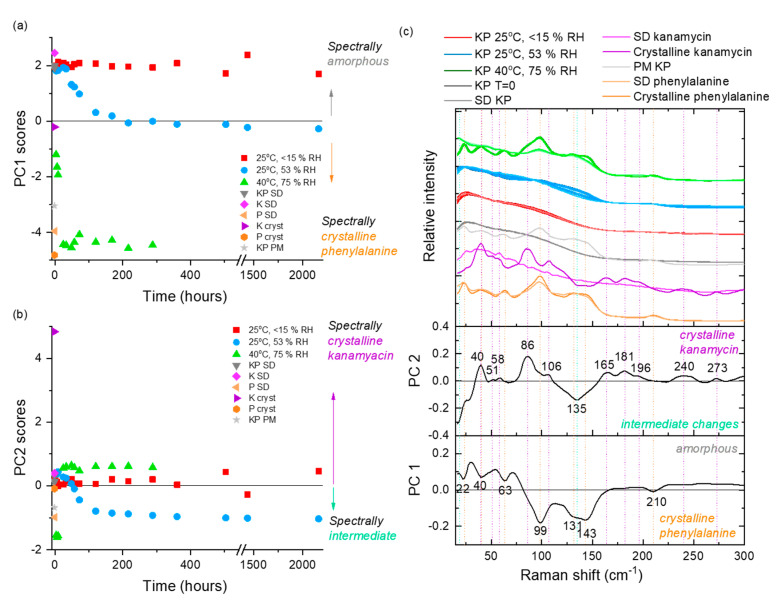
PCA analysis of the LFR spectra collected from KP samples stored under three different conditions (25 °C/<15% RH, 25 °C/53% RH and 40 °C/75% RH) over time. (**a**) PC 1 scores versus time, (**b**) PC 2 scores versus time, and (**c**) the associated loadings with comparative spectra. PM represents a physical mixture of amorphous kanamycin and crystalline phenylalanine in a 1:1 molar ratio. PC 1 accounts for 83% of the explained spectral variance, and PC 2 accounts for a further 11% explained variance. Spectra from the different storage conditions in (**c**) have slight color graduations to highlight early (lighter coloring) versus latter (darker coloring) time points.

**Figure 7:**
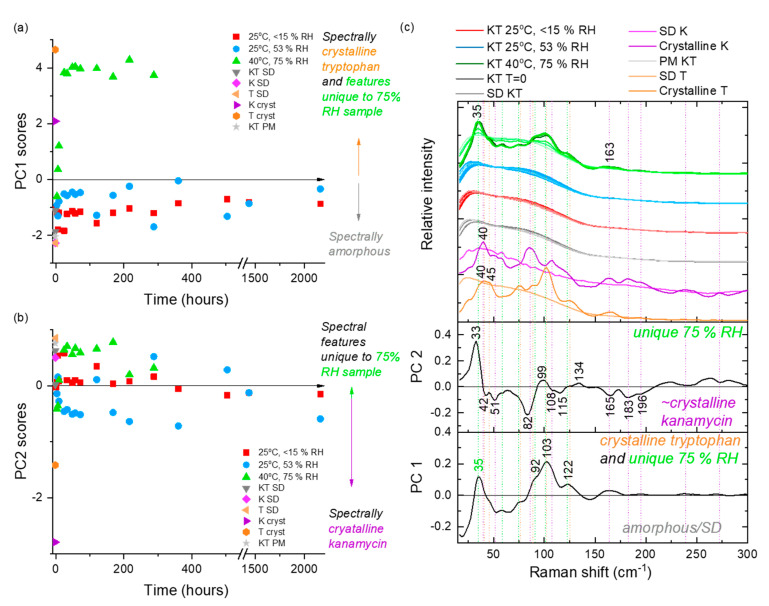
PCA analysis of the LFR spectra collected from KT samples stored under three different conditions (25 °C/<15% RH, 25 °C/53% RH and 40 °C/75% RH) over time. (**a**) PC1 scores versus time, (**b**) PC2 scores versus time, and (**c**) the associated loadings with comparative spectra. PM represents a physical mixture of amorphous kanamycin and amorphous tryptophan in a 1:1 molar ratio. PC 1 accounts for 83% of the explained spectral variance and PC 2 accounts for a further 7% explained variance. Spectra from the different storage conditions in (**c**) have slight color graduations to highlight early (lighter coloring) versus latter (darker coloring) time points.

At 25 °C/<15% RH, storage of KO formulation for 28 days decreased (*p* < 0.05) only the RF_3µm_ (Table 3). The decrease was possibly due to moisture sorption as moisture is known to increase the cohesive force between particles [70,71]. No significant change in FPF or RF_3µm_ was observed for any of the co-amorphous formulations. Spectroscopic assessment suggested the kanamycin in the KO formulation remained amorphous throughout the study duration. This was illustrated by the samples remaining in the negative PC 1 score space (Figure 5a) with the associated PC1 loadings being consistent with amorphous spectrum minus the crystalline features (Figure 5c). Similarly, all the co-amorphous formulations remained amorphous for the study duration based on the LFR spectra (Figure 6 and Figure 7 and Figures S8 and S9).

At 25 °C/53% RH, storage of KO formulation for 28 days decreased (*p* < 0.05) both the FPF and RF_3µm_ (Table 4, Figure S7), although no change in amorphicity was evident from the PCA of LFR spectra (Figure 5). The decrease in FPF was likely a result of moisture uptake by the formulation (Table 4), which facilitated particle sticking and bridging (Figure S6). However, an increase in ED (%) was observed. The increase was possibly mediated via the reduction in electrostatic charge of the particles due to humidity [72]. The peak position of the highest intensity peak in the fingerprint region of the Raman spectrum (~974 cm^−1^; associated with asymmetric stretching vibrations of sulfate moiety) gradually shifted to higher wavenumber with longer storage time, likely due to the band’s sensitivity to water (Figure S10). The trend change above 500 h (day 21) back towards a lower wavenumber may also indicate a loss of water related to the initiation of slow crystallization process (below the limit of spectroscopic detection). Similarly, moisture uptake decreased (*p* < 0.05) the FPF and RF_3µm_ of the KV formulation. The decrease was due to particle sticking via the crystallization of valine as depicted by Raman spectroscopy (Figure S8) and SEM images (Figure 4, Figure S6). The crystallization of valine occurred by 24 h, after which the formulation remained fairly spectroscopically constant. No change in FPF or RF_3µm_ was observed for the KM formulation, although methionine transformed into its crystalline counterpart (Figure S9) by 24 h. This intriguing behavior was observed as the transformation was not accompanied by any change in particle morphology, as shown by SEM images (Figure 4, Figure S6). The decrease (*p* < 0.05) in FPF and RF_3µm_ of the KP formulation was accompanied by a change in amorphicity of the formulation, which manifested into morphological changes and clustering (Figure 4, Figure S6). Gradual changes with increasing relative abundance of crystalline phenylalanine signals (negative PC 1 and negative PC 2 scores) were observed over the first 8 days, after which this system appeared spectroscopically stable (Figure 6). The FPF and RF_3µm_ of KT also decreased (*p* < 0.05), likely due to the sorption of moisture as no signs of transformation were observed (Figure 7).

At 40 °C/75% RH, formulations changed into fused masses; therefore, aerosolization evaluation was not done. Components of each formulation crystallized out into their corresponding crystalline counterparts (Figure 5, Figure 6 and Figure 7 and Figures S8 and S9). For the KO formulation, Kanamycin crystallized out within 24 h via an intermediate state (observed at 3, 6, and 9 h time-points). In LFR, the crystalline features were described by a positive PC 1 score space (Figure 5), which was consistent with that of crystalline kanamycin, whereas PC 2 described the intermediate state in negative PC 2 score space and was associated with the feature at 142 cm^−1^ (Figure 5). The intermediate state is believed to be related to the characteristic swelling behavior or “gel-like” appearance when kept at these conditions, which might promote formation of some unique but temporal structural inclusions. Spectroscopically, the KV, KM, and KP formulations exhibited a rapid crystallization of the amino acids (3–9 h) followed by the crystallization of kanamycin (24 h). The KT formulation resulted in a unique peak at 35 cm^−1^, which was not consistent with the reference crystalline features in kanamycin or tryptophan (Figure 7). It is not implausible that this could be a co-crystal; however, future work is needed to explore the origin of this feature.

For all samples the transformations were also confirmed using PXRD at the end of 3 months (Figures S11–S15). These all displayed the presence of both amino acid and kanamycin crystalline features. This was consistent with the LFR results in all instances except the KT sample where the LFR of the samples stored at 40 °C suggested a unique solid state form. However, it is worth noting that the last LFR spectra measured from the 40 °C KT samples were carried out at 12 days as opposed to 3 months for the PXRD. We cannot rule out the potential for additional changes to occur during this time period.

## 4. Conclusions

Co-amorphous formulations of kanamycin can be produced by co-spray drying with valine, methionine, phenylalanine, and tryptophan. Co-amorphization with the amino acids, except valine, significantly improves the aerosolization of kanamycin with the best aerosolization achievement with methionine. LFR spectroscopy coupled with PCA is an effective method for monitoring changes in crystallinity during storage. The kanamycin-tryptophan formulation was the most physically stable formulation, whereas the kanamycin-methionine formulation exhibited the highest aerosolization stability at 53% relative humidity, indicating a complex relationship between the physical stability and aerosolization stability of the co-amorphous systems, which remains a subject of further investigation. To the best of our knowledge, this is the first case of drug-amino acid co-amorphous spray-dried particles offering enhanced aerosolization compared to spray-dried particles containing drug alone when both were prepared using the same spray drying conditions.

## Figures and Tables

**Table 1:** Preparation and process yield of the various spray-dried particles. Values are expressed as mean ± standard deviation.

Formulation	Kanamycin (mg)	Amino Acid (mg)	Kanamycin: Amino Acid (Molar Ratio)	Final Volume (mL)	Feed Conc. (Relative to Kanamycin)	Process Yield (%), *n* = 2
Kanamycin only (**KO**)	1500	-	-	750	0.2	71.9 ± 3.7
Kanamycin-Valine (**KV**)	1500	301.5	1:1	750	0.2	70.7 ± 1.3
Kanamycin-Methionine (**KM**)	1500	384.0	1:1	750	0.2	74.4 ± 5.0
Kanamycin-Phenylalanine (**KP**)	1500	425.5	1:1	750	0.2	75.2 ± 2.2
Kanamycin-Tryptophan (**KT**)	1500	526.0	1:1	750	0.2	76.8 ± 1.3

**Table 2:** Water content and glass transition of the different spray-dried particles. Values are expressed as mean ± standard deviation.

Spray-Dried Particles	Water Content (%) (*n* = 2)	Glass Transition (°C) (*n* = 3)
Kanamycin only (KO)	7.0 ± 0.4	90.7 ± 0.9
Kanamycin-valine (KV)	4.9 ± 0.1	89.9 ± 0.2
Kanamycin-methionine (KM)	6.0 ± 1.3	90.2 ± 0.4
Kanamycin-phenylalanine (KP)	4.9 ± 0.5	90.8 ± 2.0
Kanamycin-tryptophan (KT)	5.8 ± 0.41	90.65 ± 1.0
Tryptophan		128.6 ± 0.3

**Table 3:** Summary of the emitted dose (ED) (%) and fine particle fraction (FPF) of the different formulations (kanamycin only (KO), kanamycin-valine (KV), kanamycin-methionine (KM), kanamycin-phenylalanine (KP), and kanamycin-tryptophan (KT)) on day 0 and day 28 when stored at 25 °C/<15% RH or 25 °C/53% RH. Values are expressed as mean ± standard deviation, *n* = 3. * The value is significantly (*p* < 0.05) different compared to the FPF or RF_3µm_ of KO on day 0. ** The value significantly (*p* < 0.05) decreased compared to the FPF or RF_3µm_ of the formulation on day 0.

		Day 0		Day 28—25 °C/<15% RH			Day 28—25 °C/53% RH		
	ED (%)	FPF (%)	RF_3µm_ (%)	ED (%)	FPF (%)	RF_3µm_ (%)	ED (%)	FPF (%)	RF_3µm_ (%)
KO	66.0 ± 4.2	71.1 ± 6.6	59.5 ± 6.3	68.0 ± 6.8	66.4± 5.2	44.5 ± 5.3 **	86.2 ± 3.6	60.7 ± 0.7 **	42.6 ± 0.4 **
KV	78.0 ± 3.3	74.7 ± 2.0	66.4 ± 2.0 *	74.1 ± 1.8	75.6 ± 0.6	66.5 ± 0.1	82.0 ± 4.4	68.1 ± 1.3 **	51.3 ± 1.8 **
KM	74.8 ± 1.3	83.9 ± 0.9 *	73.9 ± 1.1 *	76.1 ± 0.9	84.3 ± 1.6	74.1 ± 1.4	76.6 ± 2.2	85.6 ± 1.0	75.1 ± 2.8
KP	74.0 ± 3.6	76.8 ± 2.1 *	68.8 ± 2.0 *	74.8 ± 3.3	74.1 ± 1.0	65.4 ± 1.7	80.0 ± 4.8	68.2 ± 1.0 **	53.3 ± 0.7 **
KT	76.9 ± 2.2	78.2 ± 3.2 *	69.4 ± 2.6 *	74.5 ± 2.6	74.9 ± 0.9	66.2 ± 1.0	75.6 ± 2.6	69.9 ± 1.3 **	60.3 ± 1.2 **

**Table 4:** Summary of water content in the different formulations on day 0 and day 28 when stored at 25 °C/<15% RH or 25 °C/53% RH. Values are expressed as mean ± standard deviation.

Formulation	Day 0 *n* = 2	25 °C/<15% RH (Day 28, *n* = 2)	25 °C/53% RH (Day 28, *n* = 2)
Kanamycin only (KO)	7.0 ± 0.4	8.0 ± 0.3	12.0 ± 0.2
Kanamycin-Valine (KV)	6.0 ± 1.3	6.3 ± 0.3	10.2 ± 0.1
Kanamycin-Methionine (KM)	4.9 ± 0.1	4.9 ± 0.1	9.7 ± 0.4
Kanamycin-Phenylalanine (KP)	4.9 ± 0.5	5.1 ± 0.4	10.2 ± 0.1
Kanamycin-Tryptophan (KT)	5.8 ± 0.41	5.7 ± 0.1	10.5 ± 0.5

## Supplementary Materials

The following are available online at https://www.mdpi.com/1999-4923/12/8/715/s1, Figure S1: Representative standard curve of concentration of kanamycin, Figure S2: Representative TGA thermograms of the formulations, Figure S3: DSC thermograms. (a) Representative DSC thermogram of kanamycin only spray-dried particles. (b) Representative MDSC thermograms (reversing heat flow only) of the formulations, Figure S4: Representative MDSC thermogram of spray dried tryptophan, Figure S5: (a) View of the kanamycin sulfate monohydrate packing and different hydrogen-bonding patters along the crystallographic *a-*axis. (b) Experimental and DFT-simulated Raman spectra of crystalline kanamycin sulfate (monohydrate), Figure S6: Representative SEM images (low magnification) of the formulations during the stability study on day 0 and day 28 when stored at 25 °C/<15 % RH and 25 °C/53 % RH, Figure S7: Drug deposition behavior of the spray dried particles over 28 days when stored at different stressed conditions (25 °C/<15% RH and 25 °C/ 53% RH), Figure S8: Principal component analysis of the LFR spectra collected from KV samples stored under three different conditions (25 °C/<15% RH, 25 °C/53% RH and 40 °C/75% RH) over time, Figure S9: Principal component analysis of the LFR spectra collected from KM samples stored under three different conditions (25 °C/<15% RH, 25 °C/53% RH and 40 °C/75% RH) over time, Figure S10: Wavenumber position changes of the highest intensity peak for spray dried kanamycin only (KO) formulation kept at 25 °C/53% RH, Figure S11: Stability Study: PXRD of the kanamycin only formulation (KO) on day 0 and day 90 when kept at 25 °C/<15% RH, 25 °C/53% RH, and 40 °C/75% RH, Figure S12: Stability Study: PXRD of the kanamycin-valine formulation (KV) on day 0 and day 90 when kept at 25 °C/<15% RH, 25 °C/53% RH, and 40 °C/75% RH, Figure S13: Stability Study: PXRD of the kanamycin-methionine formulation (KM) on day 0 and day 90 when kept at 25 °C/<15% RH, 25 °C/53% RH, and 40 °C/75% RH, Figure S14: Stability Study: PXRD of the kanamycin-phenylalanine formulation (KP) on day 0 and day 90 when kept at 25 °C/<15% RH, 25 °C/53% RH, and 40 °C/75% RH, Figure S15: Stability Study: PXRD of the kanamycin-tryptophan formulation (KT) on day 0 and day 90 when kept at 25 °C/<15% RH, 25 °C/53% RH, and 40 °C/75% RH, Table S1: IR band positions (selected) in kanamycin-methionine co-amorphous system (KM), kanamycin-valine co-amorphous system (KV), kanamycin-phenylalanine co-amorphous system (KP), kanamycin-tryptophan co-amorphous system (KT), physical mixture of amorphous kanamycin and amorphous tryptophan (PM), amorphous kanamycin, amorphous tryptophan, Table S2: Summary of the particle size analysis of the formulations.
