# Supplementary material for: Co-Amorphization of Kanamycin with Amino Acids Improves Aerosolization
Source: Pharmaceutics. 2020 Jul 30;12(8):715. doi: 10.3390/pharmaceutics12080715 (PMC7465208; doi:10.3390/pharmaceutics12080715)
Supplement: Supplementary file 1 [file pharmaceutics-12-00715-s001.pdf]

pharmaceutics

MDPI

Supplementary Materials: Co-amorphization of Kanamycin with Amino acids Improves Aerosolization

Bishal Raj Adhikari, Kārlis Bērziņš, Sara J. Fraser-Miller, Keith C. Gordon and Shyamal C. Das

Table S1 IR band positions (selected) in kanamycin-methionine co-amorphous system (KM), kanamycin-valine co-amorphous system (KV), kanamycin-phenylalanine co-amorphous system (KP), kanamycin-tryptophan co-amorphous system (KT), physical mixture of amorphous kanamycin and amorphous tryptophan (PM), amorphous kanamycin, amorphous tryptophan.

KM [IR bands(cm^{-1})]	KV [IR bands(cm^{-1})]	KP [IR bands(cm^{-1})]	KT [IR bands(cm^{-1})]	PM [IR bands(cm^{-1})]	Amorphous Kanamycin [IR bands(cm^{-1})]	Amorphous Tryptophan [IR bands(cm^{-1})]
			427	423		424 (Benzene ring deformation vibration)
			460	457		461 (Pyrrole ring deformation vibration)
609	609	609	608	608	608 (Sulfate ion bending)	
			749	742		740 (Benzene/ pyrrole ring deformation vibration)
1031	1030	1031	1033	1032	1029 (Sulfate ion stretching)	
1338	1332	1336	1342	1340	1337 (C-H bending)	1339 (C-H bending)
			1402	1397		1396 (-COOH sym. stretching)
1521	1520	1521	1522	1519	1528 ($-NH_2/NH_3^+$ sym. bending)	1490 ($-NH_2$ sym. bending)
1602	1600	1602	1602	1607	1605 ($-NH_2/NH_3^+$ asym. bending)	1601 (-COOH asym. stretch/ $-NH_2$ asym. bend/ Benzene ring stretching)

Table S2 Summary of the particles size analysis of the formulations. Values are expressed as mean ± standard deviation.

**Formulation**	**SEM (µm) (n=300)**
Kanamycin Only (KO)	1.1 ± 0.5
Kanamycin-valine (KV)	1.1 ± 0.5
Kanamycin-methionine (KM)	1.0 ± 0.5
Kanamycin-phenylalanine (KP)	1.2 ± 0.5
Kanamycin-tryptophan (KT)	1.1 ± 0.6

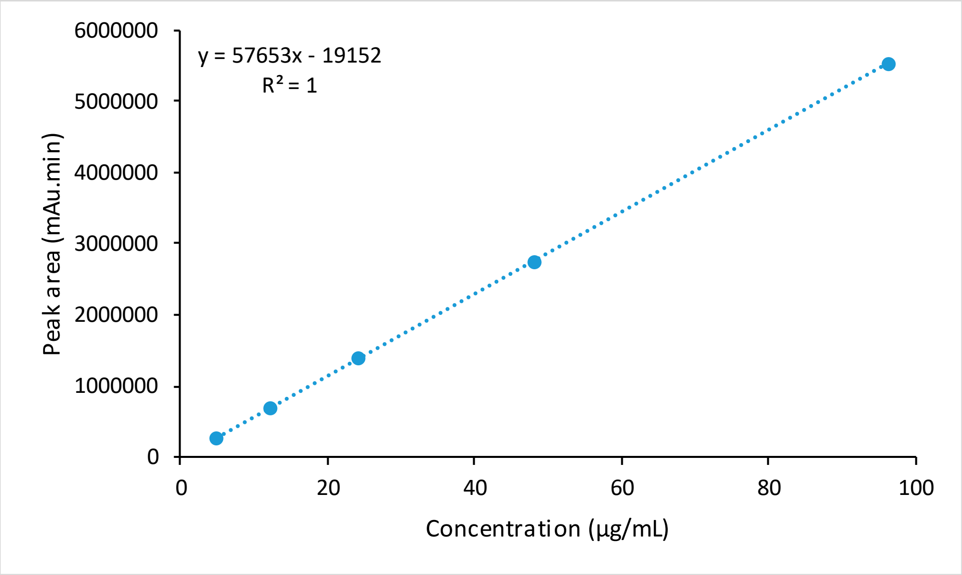

Figure S1 Representative standard curve of concentration of kanamycin (R^2= 0.99692).

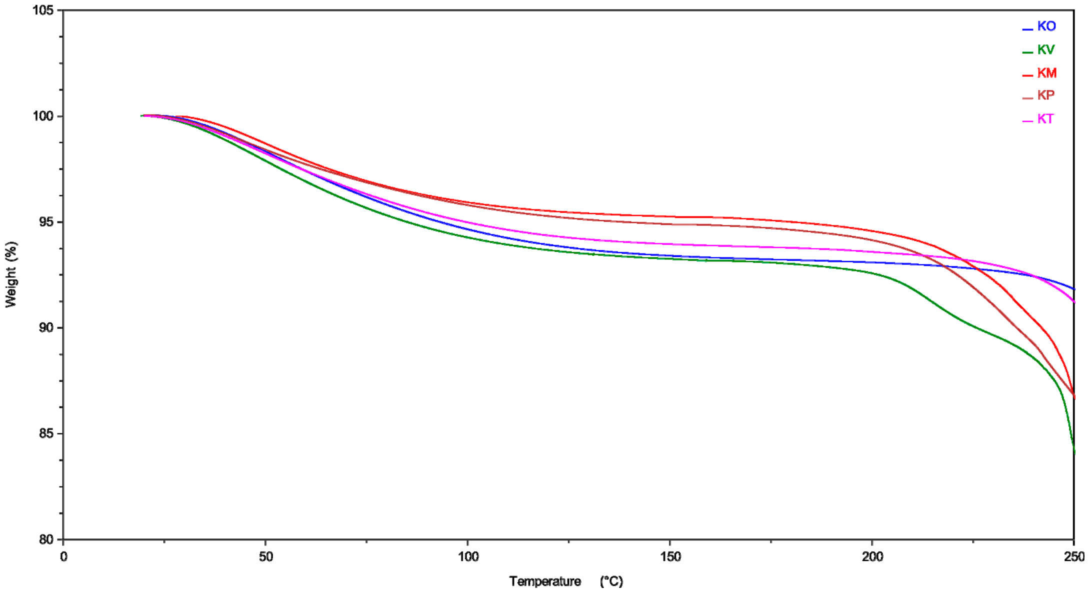

Figure S2 Representative TGA thermograms of the formulations [kanamycin only (KO), kanamycin-valine (KV), kanamycin-methionine (KM), kanamycin-phenylalanine (KP), and kanamycin-tryptophan (KT)].

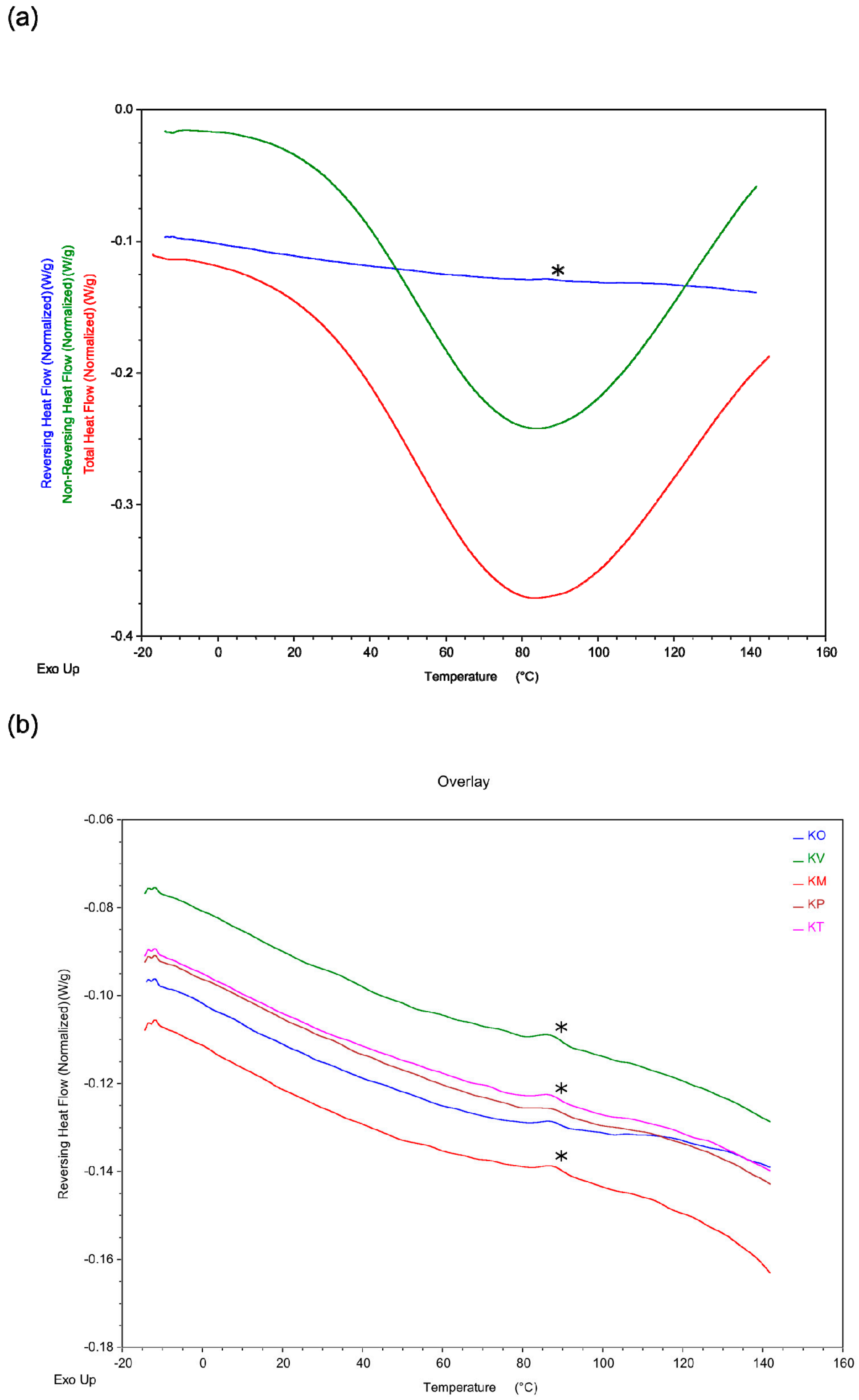

Figure S3 DSC thermograms. (a) Representative DSC thermogram of kanamycin only spray-dried particles. (b) Representative MDSC thermograms (reversing heat flow only) of the formulations [kanamycin only (KO), kanamycin-valine (KV), kanamycin-methionine (KM), kanamycin-phenylalanine (KP), and kanamycin-tryptophan (KT)]. [Asterisk (*) shows the step change associated with glass transition temperature.]

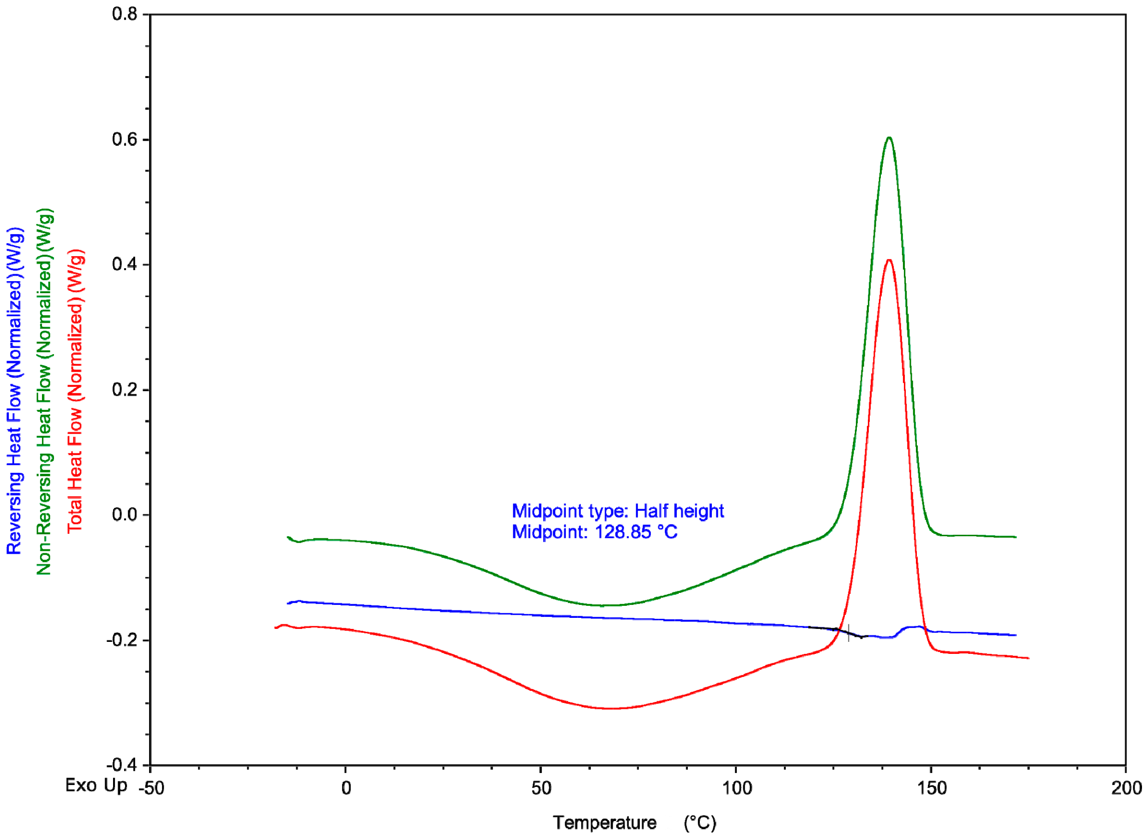

Figure S4 Representative MDSC thermogram of spray-dried tryptophan.

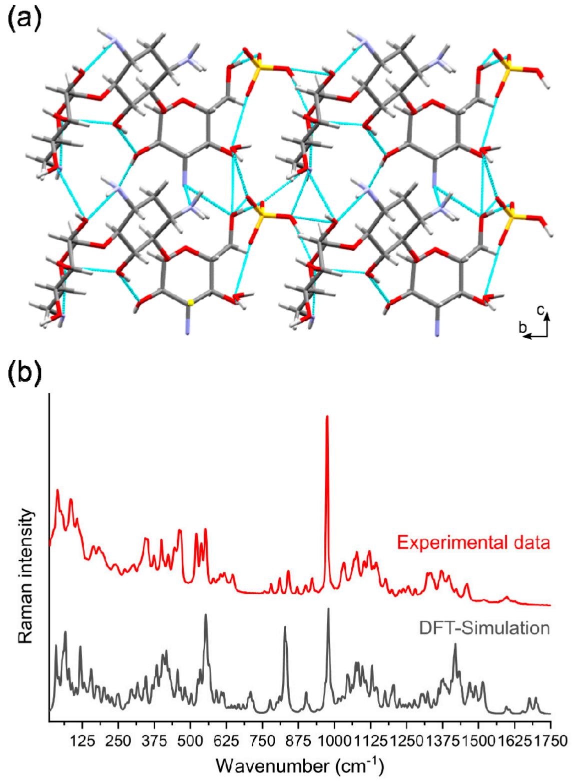

Figure S5 (a) View of the kanamycin sulfate monohydrate packing and different hydrogen-bonding patters along the crystallographic *a*-axis. (b) Experimental and DFT-simulated Raman spectra of crystalline kanamycin sulfate (monohydrate).

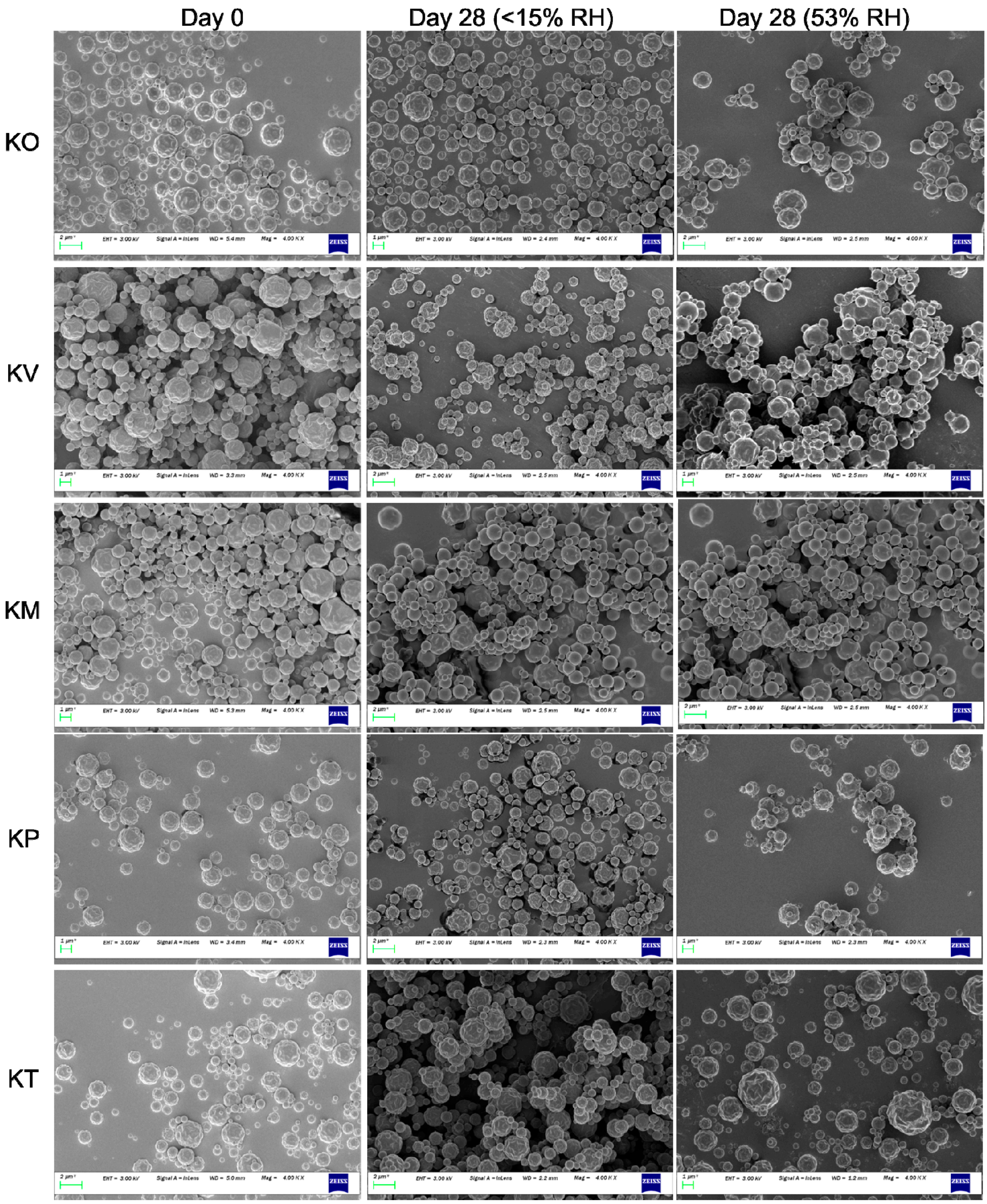

Figure S6 Representative SEM images (low magnification) of the formulations [kanamycin only (KO), kanamycin-valine (KV), kanamycin-methionine (KM), kanamycin-phenylalanine (KP), and kanamycin-tryptophan (KT)] during the stability study on day 0 and day 28 when stored at 25 °C/<15 % RH and 25 °C/53 % RH.

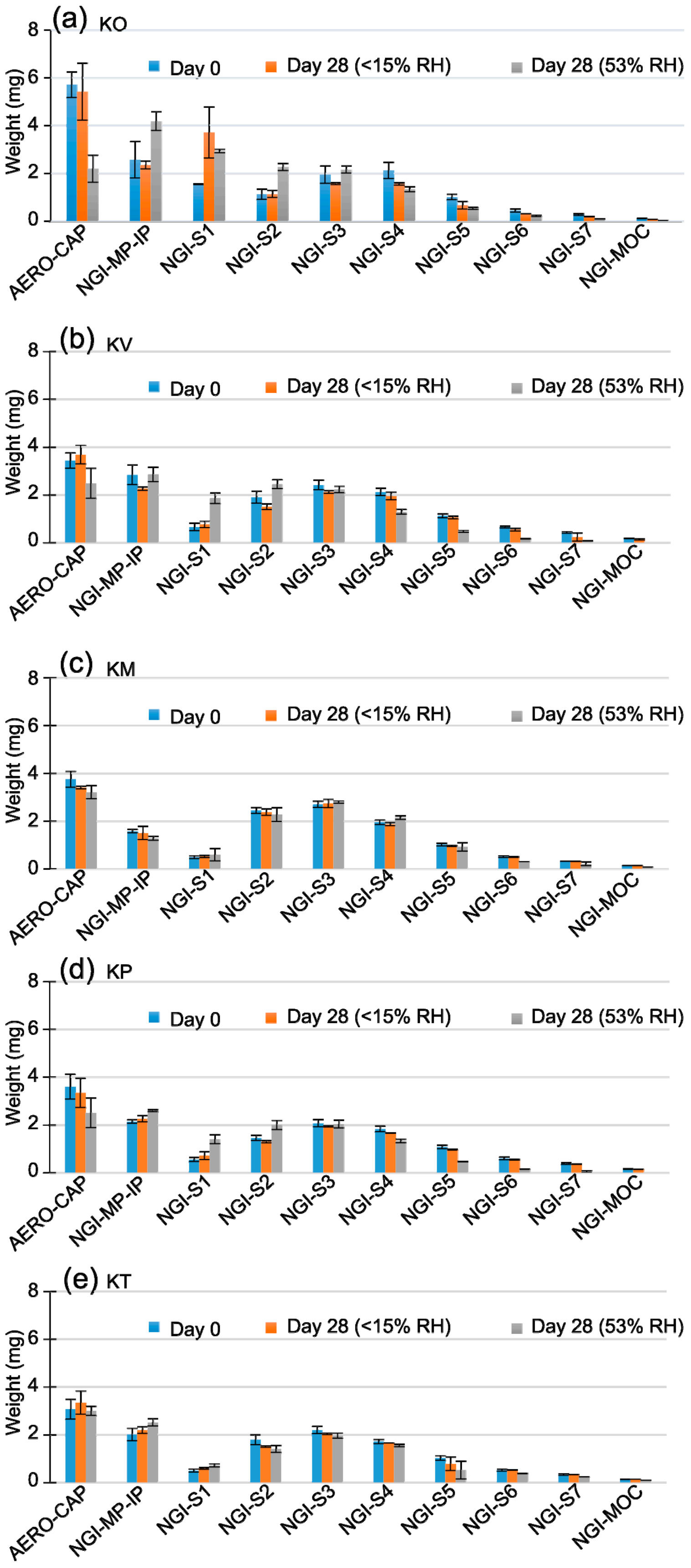

Figure S7 Drug deposition behavior of the spray-dried particles over 28 days when stored at different stressed conditions (25 °C/<15% RH and 25 °C/ 53% RH) for (a) kanamycin only (KO), (b) kanamycin-valine (KV), (c) kanamycin-methionine (KM), (d) kanamycin-phenylalanine (KP), and (e) kanamycin-tryptophan (KT) formulations.

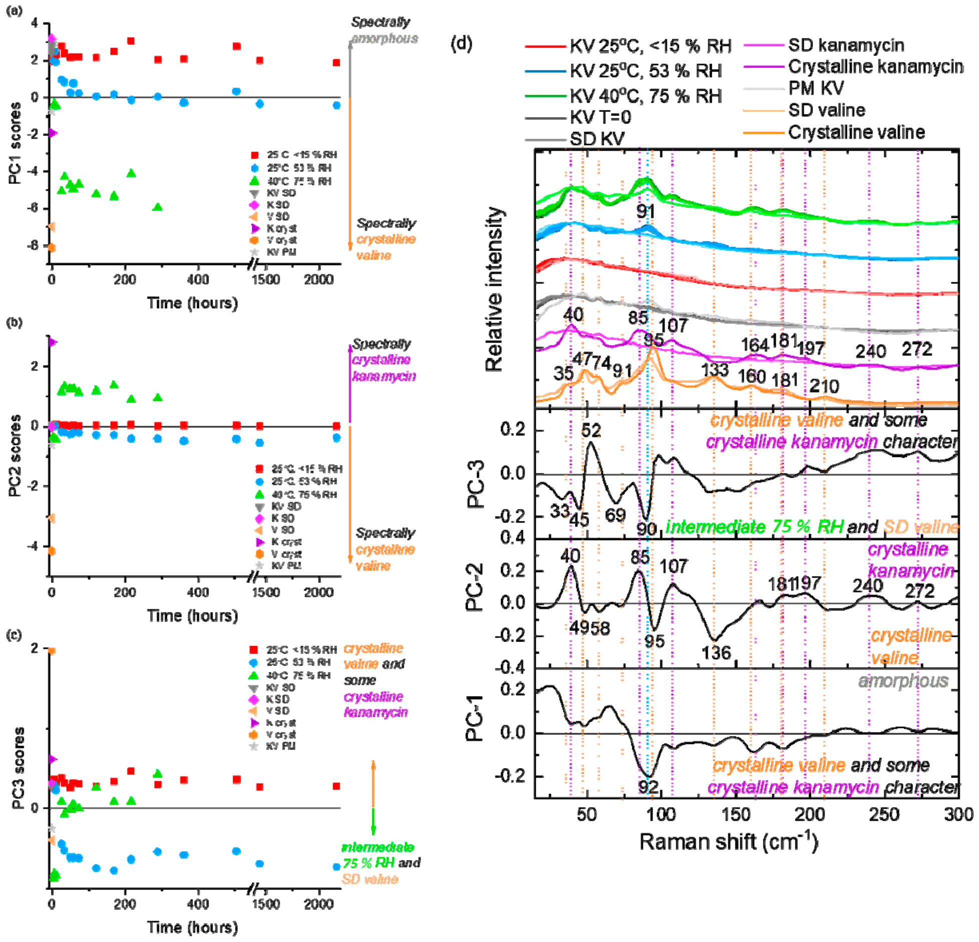

Figure S8 Principal component analysis of the LFR spectra collected from KV samples stored under three different conditions (25 °C/<15% RH, 25 °C/53% RH and 40 °C/75% RH) over time. (a) PC 1 scores versus time, (b) PC 2 scores versus time, (c) PC3 scores versus time and (d) loadings with comparative spectra. PC 1 accounts for 86% of the explained spectral variance, PC 2 accounts for a further 9% and PC 3 a further 3% explained variance. PM represents physical mixture of amorphous kanamycin and crystalline valine in 1:1 molar ratio. Spectra from the different storage conditions in (c) have slight color graduations to highlight early (lighter coloring) versus latter (darker coloring) time points.

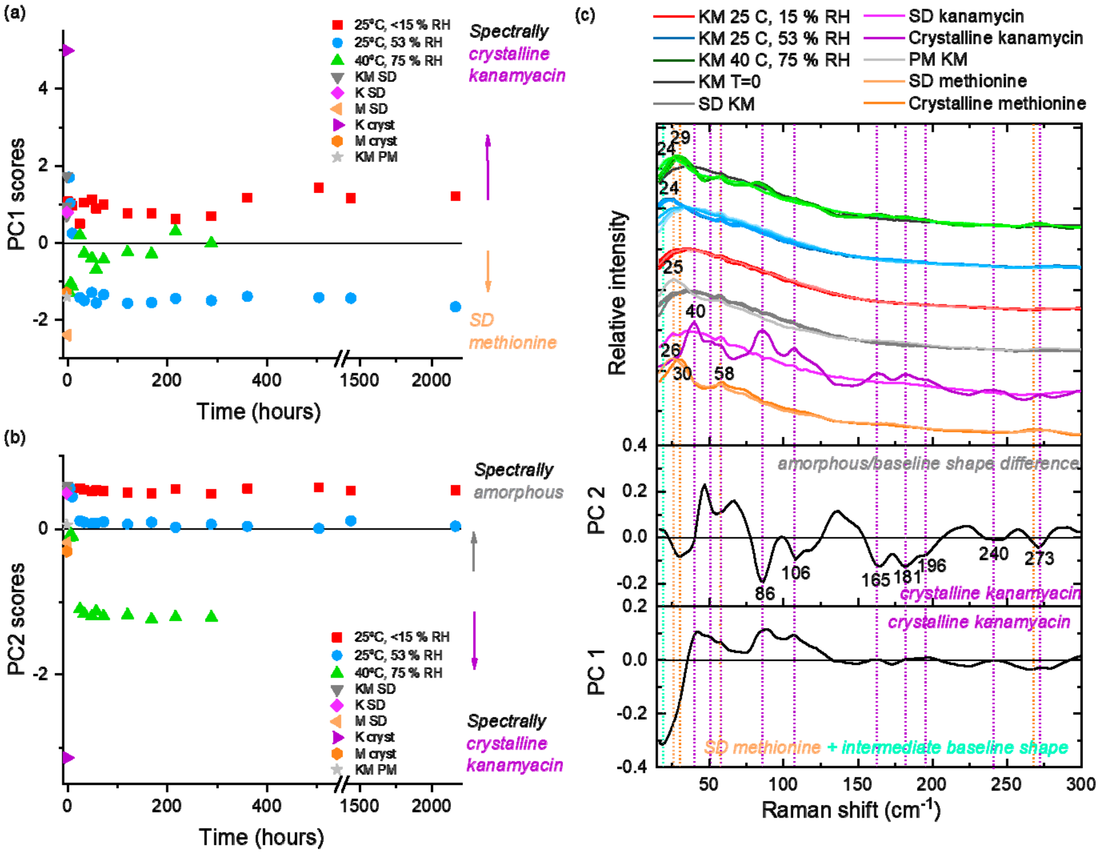

Figure S9 Principal component analysis of the LFR spectra collected from KM samples stored under three different conditions (25 °C/<15% RH, 25 °C/53% RH and 40 °C/75% RH) over time. (a) PC 1 scores versus time, (b) PC 2 scores versus time, and (c) loadings with comparative spectra. PC 1 accounts for 67% of the explained spectral variance, PC 2 accounts for a further 21% explained variance**.** PM represents physical mixture of amorphous kanamycin and crystalline methionine in 1:1 molar ratio. Spectra from the different storage conditions in (c) have slight color graduations to highlight early (lighter coloring) versus latter (darker coloring) time points.

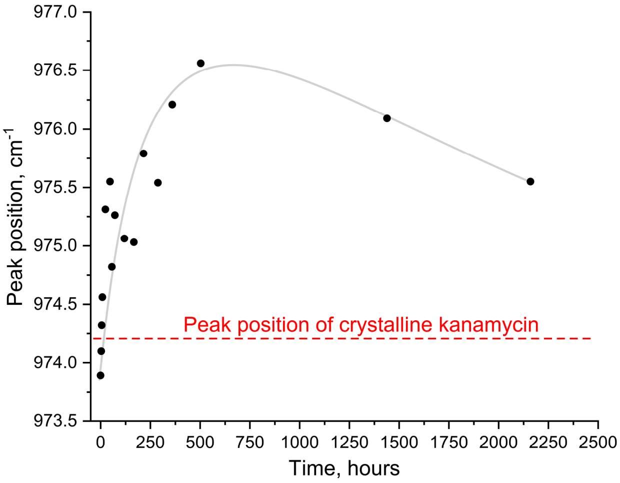

Figure S10 Wavenumber position changes of the highest intensity peak for spray-dried kanamycin only (KO) formulation kept at 25 °C/53% RH. The line is drawn to assist in visualizing the trend.

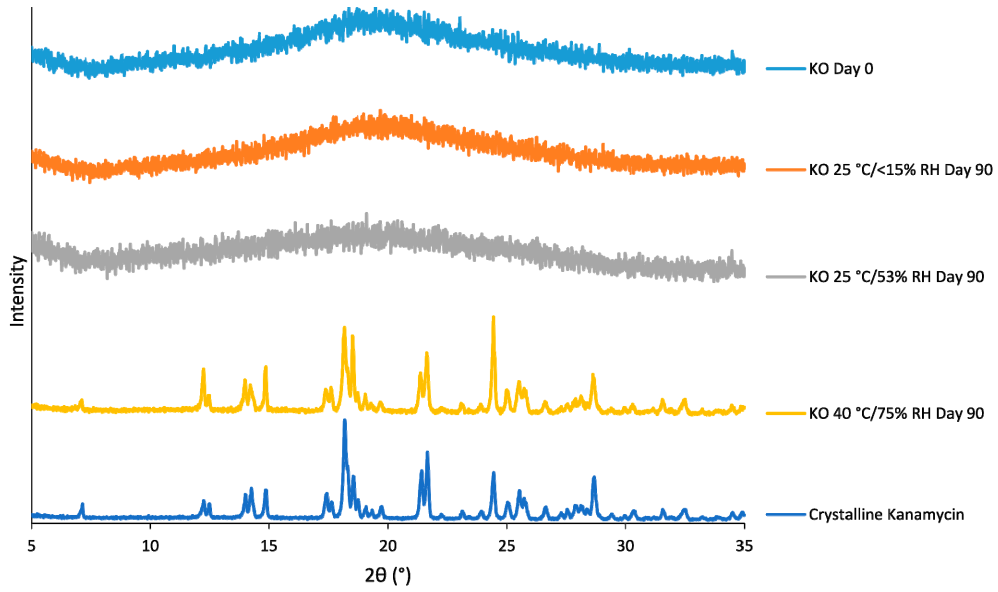

Figure S11 Stability Study: PXRD of the kanamycin only formulation (KO) on day 0 and day 90 when kept at 25 °C/<15% RH, 25 °C/53% RH, and 40 °C/75% RH.

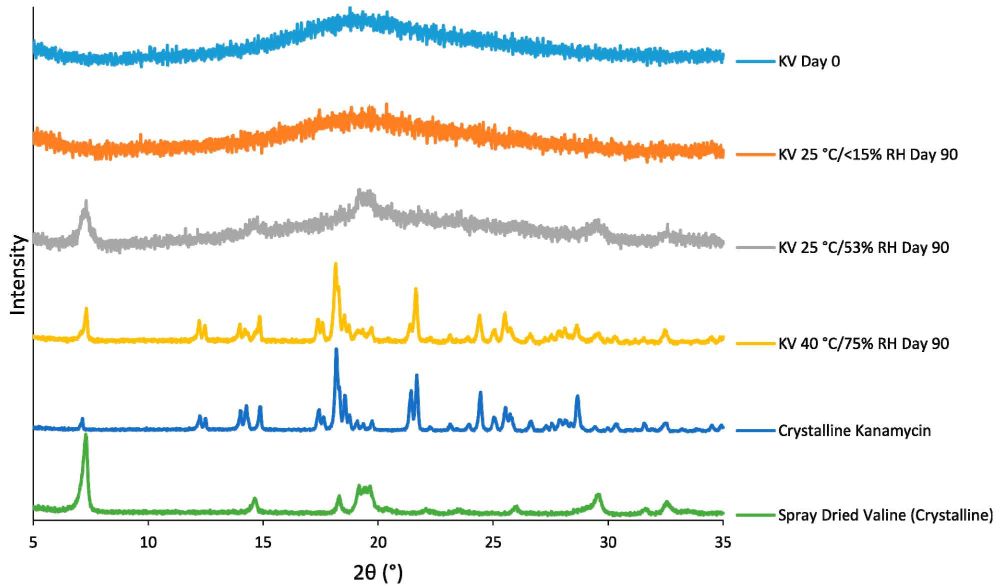

Figure S12 Stability Study: PXRD of the kanamycin-valine formulation (KV) on day 0 and day 90 when kept at 25 °C/<15% RH, 25 °C/53% RH, and 40 °C/75% RH.

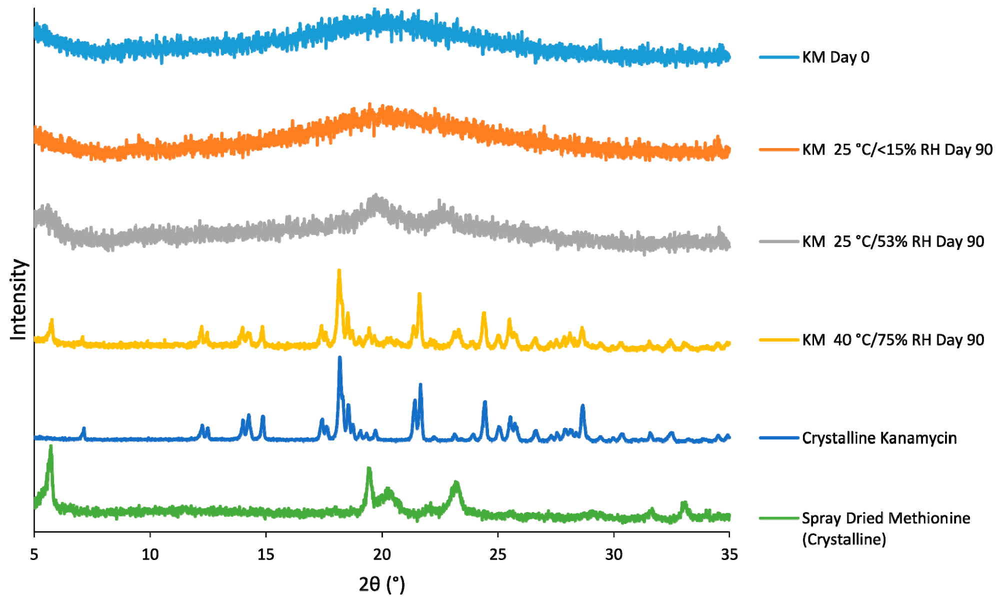

Figure S13 Stability Study: PXRD of the kanamycin-methionine formulation (KM) on day 0 and day 90 when kept at 25 °C/<15% RH, 25 °C/53% RH, and 40 °C/75% RH.

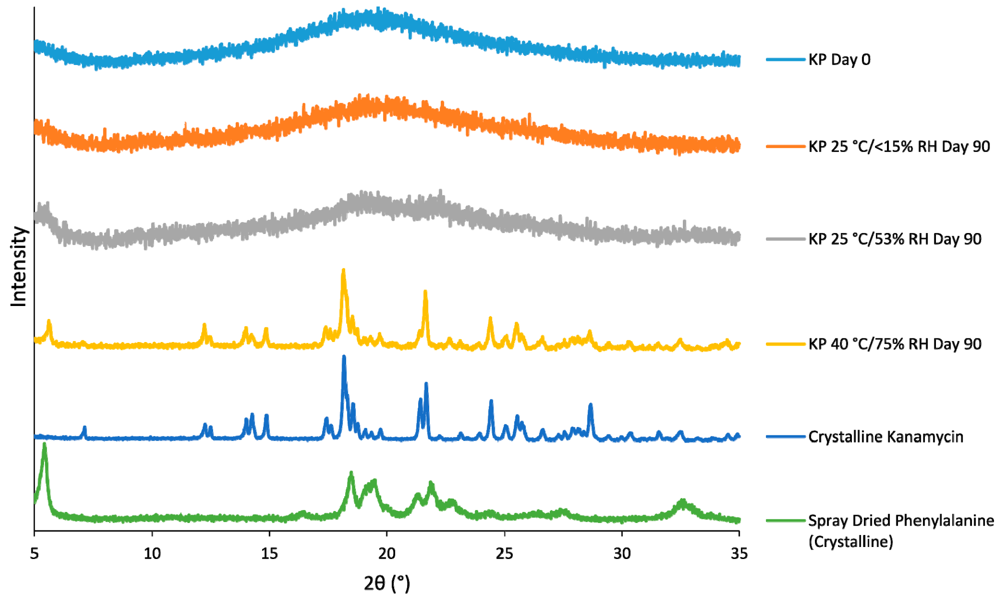

Figure S14 Stability Study: PXRD of the kanamycin-phenylalanine formulation (KP) on day 0 and day 90 when kept at 25 °C/<15% RH, 25 °C/53% RH, and 40 °C/75% RH.

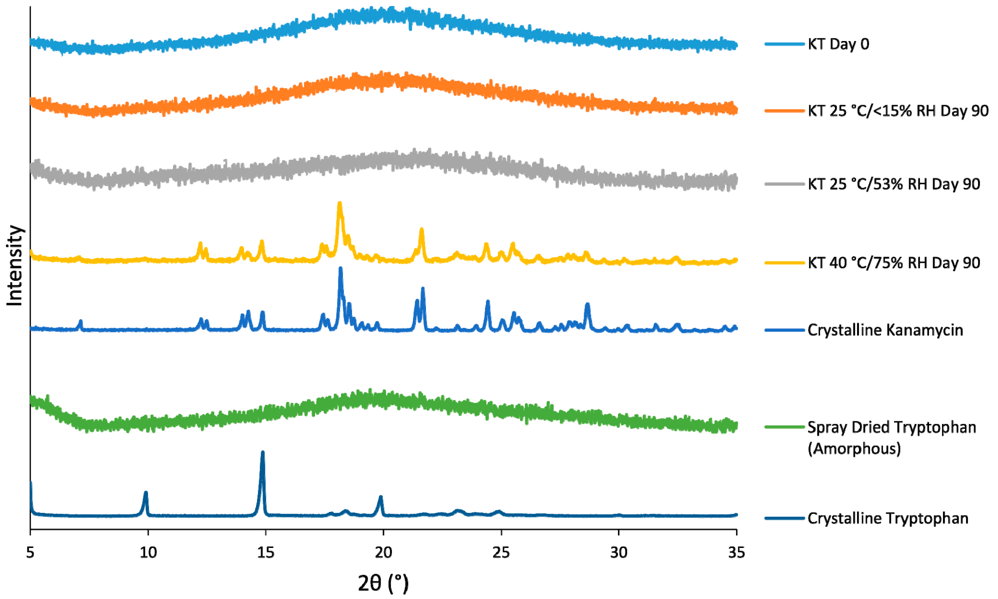

Figure S15 Stability Study: PXRD of the kanamycin-tryptophan formulation (KT) on day 0 and day 90 when kept at 25 °C/<15% RH, 25 °C/53% RH, and 40 °C/75% RH.
